# Transcriptomic and Proteomic Analysis of Monkeypox Virus A5L-Expressing HEK293T Cells

**DOI:** 10.3390/ijms26010398

**Published:** 2025-01-05

**Authors:** Mingzhi Li, Jiaqi Xiong, Hao Zhou, Jing Liu, Chenyi Wang, Mengle Jia, Yihao Wang, Nannan Zhang, Yanying Chen, Tao Zhong, Zhicheng Zhang, Ruiying Li, Yuxin Zhang, Yunli Guo, Qi Peng, Lingbao Kong

**Affiliations:** 1 Institute of Pathogenic Microorganism, Jiangxi Agricultural University, Nanchang 330000, China; 2 Nanchang City Key Laboratory of Animal Virus and Genetic Engineering, Nanchang 330000, China; 3 College of Bioscience and Engineering, Jiangxi Agricultural University, Nanchang 330000, China

**Keywords:** transcriptome, proteome, monkeypox virus, A5L

## Abstract

Monkeypox (MPOX) is a zoonotic viral disease caused by the Monkeypox virus (MPXV), which has become the most significant public health threat within the *Orthopoxvirus* genus since the eradication of the Variola virus (VARV). Despite the extensive attention MPXV has garnered, little is known about its clinical manifestations in humans. In this study, a high-throughput RNA sequencing (RNA-seq) and liquid chromatography-tandem mass spectrometry (LC-MS/MS) approach was employed to investigate the transcriptional and metabolic responses of HEK293T cells to the MPXV A5L protein. RNA-seq analysis identified a total of 1473 differentially expressed genes (DEGs), comprising 911 upregulated and 562 downregulated genes. Additionally, LC-MS/MS analysis revealed 185 cellular proteins with significantly altered abundance ratios that interact with the A5L protein. Here, we perform Gene Ontology (GO) and Kyoto Encyclopedia of Genes and Genomes (KEGG) analysis of the transcriptome and proteome signatures of MPXV A5L-expressing HEK293T cells to gain insights into the virus proteins-host interplay. Transcriptomic analysis revealed that transfection of the MPXV A5L protein modulated genes primarily associated with the cell cycle, ribosome, and DNA replication. Proteomic analysis indicated that this protein predominantly interacted with host ribosomal proteins and cytoskeletal proteins. The combination of transcriptomic and proteomic analysis offers new perspectives for understanding the interaction between pathogens and hosts. Our research emphasizes the significant role of MPXV A5L in facilitating viral internalization and assembly, as well as its impact on the host’s translation system.

## 1. Introduction

Monkeypox is a viral zoonotic infectious disease caused by the Monkeypox virus [1,2,3]. In 1958, MPXV was first identified during a vesicular disease outbreak in captive African monkeys that had been shipped to Copenhagen, Denmark, from Central and West Africa [4]. Sporadic cases of MPXV in humans were first identified in the 1970s in several African countries [3]. Subsequently, sporadic outbreaks of MPOX occurred in Africa, but these events received limited international attention. Between 2003 and 2022, there was an increase in the number of MPOX cases reported outside the epidemic countries, and the risk of human-to-human transmission of the virus remains a persistent threat [3]. The largest MPXV outbreak of the 21st century occurred in 2022, leading to epidemics in numerous countries [4]. The rapid spread of MPXV in 2022 led to the World Health Organization (WHO) declaring a Public Health Emergency of International Concern (PHEIC) [5]. As of 1 July 2024, more than 97,000 laboratory-confirmed human cases of MPOX had been reported (https://www.cdc.gov/mpox/data-research/cases/index.html, accessed on 1 July 2024).

MPXV is a large double-stranded DNA virus belonging to the *Orthopoxvirus* genus within the *Poxviridae* family, which also includes VARV, vaccinia virus (VACV), and Cowpox virus (CPXV) [1,3]. A distinctive feature used to distinguish VACV infections from MPXV infections is the swelling of the maxillary, cervical, or inguinal lymph nodes (lymphadenopathy) [3,5,6,7]. It contains a double-stranded DNA genome, which is approximately 197 kb in length and includes at least 190 open reading frames (ORFs) [5,8,9]. Phylogenetically, historic MPXV isolates cluster into two main clades, designated Clade I (Ia and Ib) and Clade II (II a and II b) [10,11,12]. Clade I viruses are considered more virulent and transmissible compared to Clade II viruses [13]. The viruses responsible for the 2022 epidemic belong to subclade IIb, a lineage that has been circulating in Nigeria since at least 1971 [3,10,14,15,16]. MPXV possesses a double-layered cell membrane and exhibits a brick-shaped morphology. It utilizes its double-stranded DNA genome to replicate within the host’s cytoplasm [17]. Although MPXV is a DNA virus, its entire lifecycle occurs in the cytoplasm of infected cells, where a variety of proteins essential for the replication machinery are encoded by ORFs within the MPXV genome [18]. Half of these ORFs are essential for viral replication and morphogenesis and are well conserved among other poxviruses [17,19]. The remaining half comprises “accessory” genes involved in immunomodulation, pathogenesis, and host tropism [17,19].

The A5L is 846 bp in length and encodes a 39 kD size A5L protein, which is a highly antigenic protein found in the viral core. Upton C et al.’s study reveals that MPXV A5L possesses gene or protein homologs (A4L) within the *Chordopoxvirinae* family, including A4L of Vaccinia virus (Copenhagen strain), A5L of the VACV WR strain (ATCC VR-119), and CPXV136 of Cowpox virus (Brighton strain) [20,21,22]. The research results on the MPXV A5L are limited. The protein is conserved in many *Chordopoxvirinae* subfamily (vaccinia viruses, horsepox virus, and cowpox virus, etc.), which means that the study of the A4L of *Chordopoxvirinae* subfamily could inform our study of MPXV A5L [21]. The VACV 39 kDa protein, the product of the *A4L* gene, is membrane associated and encapsidated into the virion [23]. A4L protein is phosphorylated in vivo and serves as a substrate for the F10L kinase in vitro. Its phosphorylation appears to be modulated by the expression of the viral H1 phosphatase [23]. Immunoelectron microscopy has revealed that antibodies against A4L protein decorate the interior of intracellular virion (IV) but not the nucleoid. Within mature virions (MV), these antibodies appear to localize to a region between the core and the membrane [23]. Pulse-chase and immunoprecipitation experiments have demonstrated that the 39 kDa protein interacts with p4a, which is encoded by the *A10L* gene and serves as the precursor to the most abundant virion protein [24]. This interaction is maintained with the processed 4a form that arises during virion maturation [24]. The colocalization of VACV WR A5L with replicated DNA in the virosomes and its copurification with nuclease activity has been reported [22]. This suggested a possible role for MPXV A5L during virus assembly.

In our research, to acquire transcriptomic information and identify interactions between MPXV-A5L protein and host proteins, HEK293T cells were transfected with either the pCAGGS-HA-A5L plasmid or the control pCAGGS-HA plasmid. Cells were harvested 24 h post-transfection and subjected to either RNA-seq or LC-MS/MS. GO and KEGG enrichment analyses were conducted to examine DEGs and potential host-interacting proteins identified in HEK293T cells overexpressing the A5L protein.

Our dataset highlights multiple signaling pathways and host proteins affected during MPXV A5L expression, thereby providing novel insights into understanding the pathogen-host interaction and aiding in the rational design of both virus- and host-directed therapies.

## 2. Results

### 2.1. Sequence Analysis of MPXV-A5L

**Figure 1:**
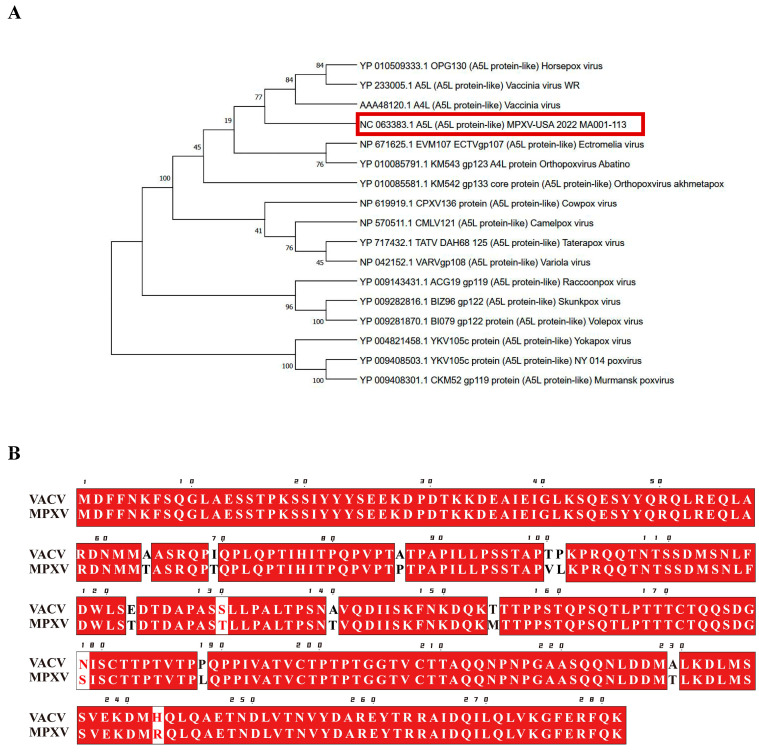
Amino acid sequence analysis of VACV-A4L and MPXV-A5L. (**A**) Molecular phylogenetic analysis of poxvirus A4L and MPXV A5L (highlighted in a red box). The tree with the highest log likelihood (−3224.27) is shown. The percentage of trees in which the associated taxa clustered together is indicated next to the branches. (**B**) Analysis of amino acid sequence identity between VACV-A4L (GeneBank: AAA48120.1) and MPXV-A5L (GeneBank: NC_063383.1). Red box with white characters indicates complete amino acid strict identity, white box with red characters indicates amino acid similarity, and black font on a white background indicates complete amino acid divergence.

To investigate the conservation of A5L among poxviruses, we conducted a phylogenetic analysis of MPXV-A5L (Figure 1A). We identified poxvirus proteins with amino acid sequences similar to MPXV-A5L, constructed a phylogenetic tree, and found that most of these proteins belong to *Chordopoxvirinae* (Figure 1A). Phylogenetic analysis revealed that MPXV-A5L and VACV-A5L exhibit shorter divergence times and closer evolutionary relationships compared to other poxvirus proteins (Figure 1A). When comparing the homology between MPXV-A5L (GeneBank: NC_063383.1) and VACV-A4L (GeneBank: AAA48120.1), we found that the two proteins share 95% sequence identity (Figure 1B).

### 2.2. Plasmid Construction and Immunoblot Analyses of MPXV A5L Protein

**Figure 2:**
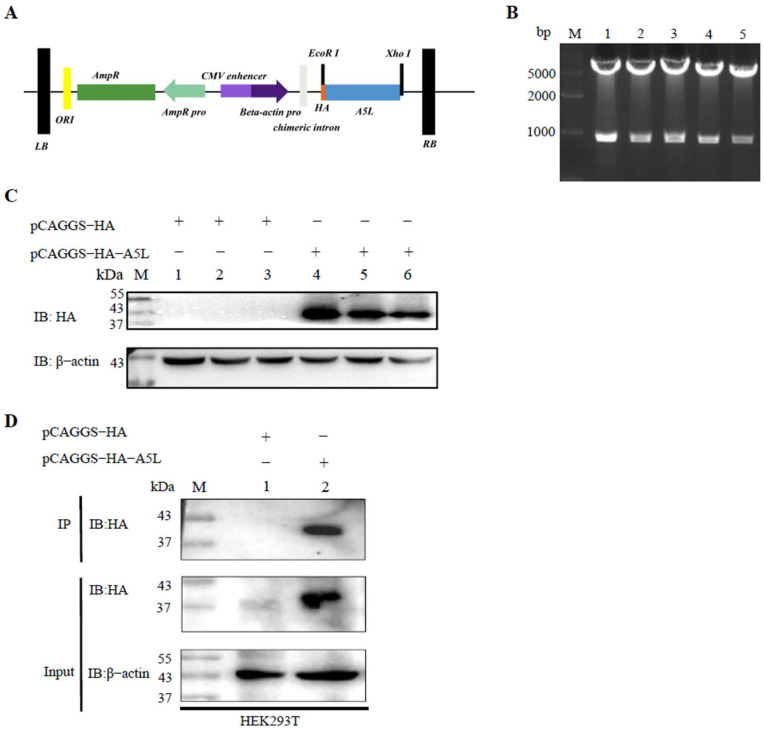
Construction and expression of MPXV A5L in HEK293T Cells. (**A**) Schematic representation of the cloned fragment. (**B**) Double digestion of the pCAGGS-HA-A5L plasmid. Lanes 1–5: The pCAGGS-HA-A5L recombinants were digested with EcoRI and XhoI. (**C**) Expression of MPXV A5L protein in HEK293T cells was followed by immunoblot analysis using anti-HA and anti-β-actin antibodies. M: 180 kDa protein molecular weight marker (8–180 kDa). Lanes 1–3: Cells transfected with pCAGGS-HA. Lanes 4–6: Cells transfected with pCAGGS-HA-A5L. (**D**) IP and whole-cell lysates (Input) used for immunoprecipitation were detected by immunoblotting with an anti-HA antibody. Lane 1: Cells transfected with pCAGGS-HA plasmid. Lane 2: Cells transfected with pCAGGS-HA-A5L plasmid.

The pCAGGS-HA-A5L plasmid was constructed and verified by DNA agarose gel electrophoresis. The sequences “GAATTC” and “CTCGAG” at both ends of *A5L* represent EcoRⅠ and XhoⅠ cleavage sites, respectively (Figure 2A). Double digestion analysis confirmed the presence of two expected bands on the gel: one at approximately 5000 bp, representing the pCAGGS-HA vector, and another at 846 bp, representing the *A5L* gene (Figure 2B). Subsequently, the pCAGGS-HA-A5L plasmid was transfected into HEK293T cells, and the expression of the A5L protein was detected using an anti-HA antibody. Immunoblot results revealed three distinct 39 kDa bands corresponding to the A5L protein, confirming successful expression of the pCAGGS-HA-A5L plasmid in 293T cells (Figure 2C). Samples from immunoprecipitation (IP) and whole-cell lysate (WCL) after anti-HA incubation (Input) were subjected to immunoblot analyses, revealing a 39 kDa band corresponding to the A5L protein in pCAGGS-HA-A5L-transfected HEK293T cells (IP and Input) (Figure 2D). These results indicate that MPXV A5L protein was effectively captured by the anti-HA agarose conjugate.

### 2.3. Identification of DEGs

**Figure 3:**
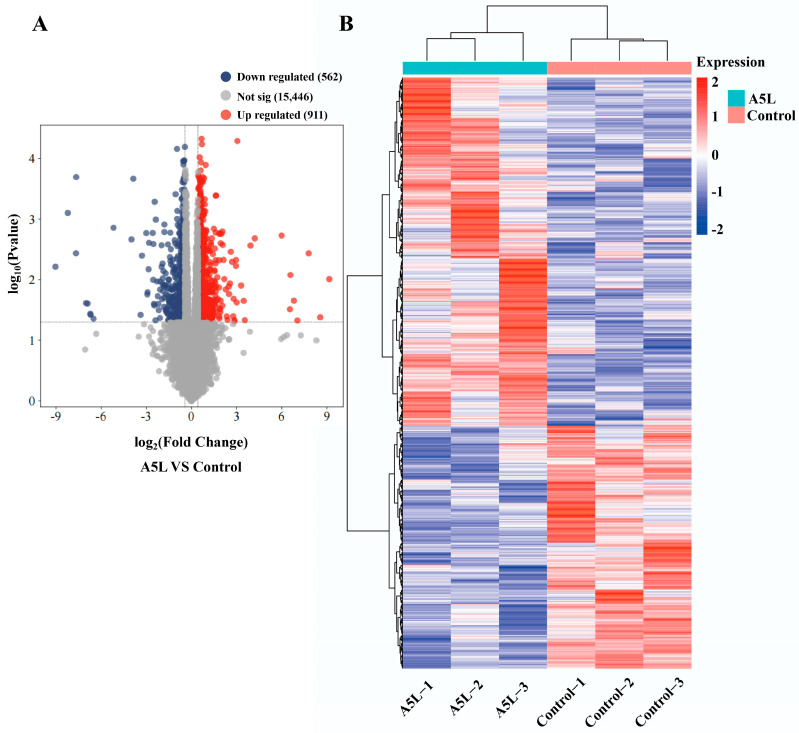
Identification of DEGs. (**A**) Volcano plot of DEGs. The vertical axis represents the significance (−log10 *p*-value), while the horizontal axis represents the log2 fold change in gene expression between the experimental and control groups. Downregulated genes by green dots, upregulated genes are indicated by red dots, and the threshold lines for DEGs screening criteria are shown as gray dashed lines. (**B**) Thermal polymerization map of DEGs. The left vertical axis represents the results of cluster analysis, while the horizontal axis denotes the sample names. The right vertical axis indicates gene names. The red color in the middle of the heatmap represents high expression, and the blue color represents low expression.

To assess the effects of the A5L protein on the transcriptome of HEK293T cells, we analyzed cellular transcriptome changes induced by A5L using HTSeq. Compared with HEK293T cells transfected with the pCAGGS-HA vector, a total of 1473 DEGs were identified in cells transfected with the pCAGGS-HA-A5L construct, including 911 upregulated and 562 downregulated genes (Figure 3A). Heatmap data revealed distinct differences in gene expression between the A5L group and the control group (Figure 3B). These findings indicate that A5L protein expression can significantly affect multiple genes and trigger substantial cellular responses.

### 2.4. GO and KEGG Analysis of DEGs

**Figure 4:**
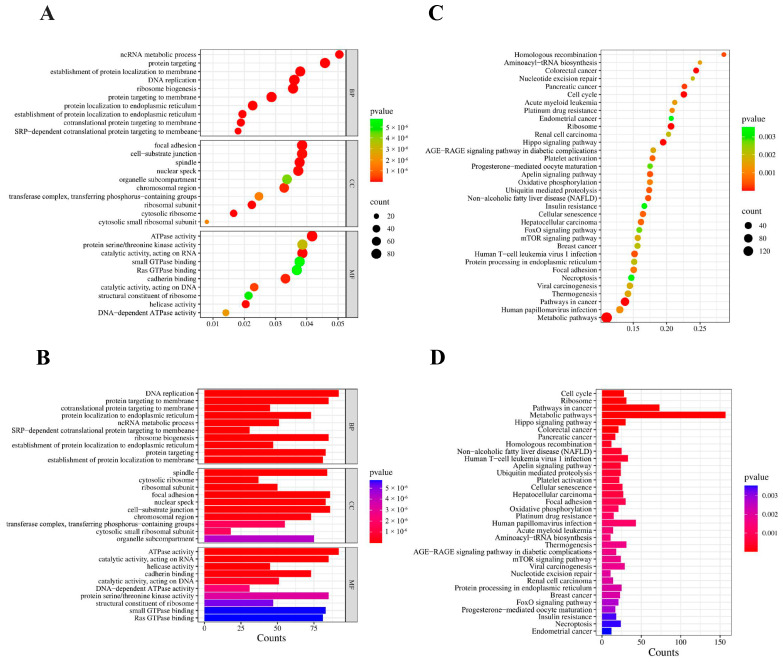
Functional enrichment analysis of DEGs. (**A**) The scatter plot and (**B**) the histogram of GO analysis. (**C**) The scatter plot and (**D**) the histogram of KEGG analysis. The vertical axis represents the top 30 or 35 most significant terms, while the horizontal axis represents the gene ratio (**A**,**C**) or count (**B**,**D**). Gene ratio: The ratio of DEGs numbers to background gene numbers. Count: The number of DEGs. *p*-value: An indicator of term significance; smaller *p*-values indicate greater significance.

GO analysis was performed to investigate the potential biological functions of the DEGs. The results indicated that the DEGs were categorized into three main groups: biological process (BP, 280 GO terms), cellular component (CC, 51 GO terms), and molecular function (MF, 20 GO terms). The top 30 enriched GO terms were presented in Figure 4A,B. BP including DNA replication (GO:0006260), protein targeting the membrane (GO:0006612) and ribosome biogenesis (GO:0042254) were highly enriched. In the CC category, the primary enriched terms included cytosolic ribosome (GO:0022626), ribosomal subunit (GO:0044391), and focal adhesion (GO:0005925). Within the MF category, A5L were predominantly associated with ATPase activity (GO:0016887), ribosomal structural components (GO:0003735), and catalytic activities targeting RNA (GO:0140098) (*p* < 0.01). These pathways also exhibited smaller adjusted *p*-values (padj) and a lower probability of false positives. The KEGG pathway enrichment analysis showed that the Cell cycle (hsa04110), Ribosome (hsa03010), and mTOR signaling pathway (hsa04150) were statistically significantly enriched (Figure 4C,D).

### 2.5. Varification of the RNA-Seq Data Through RT-qPCR

**Figure 5:**
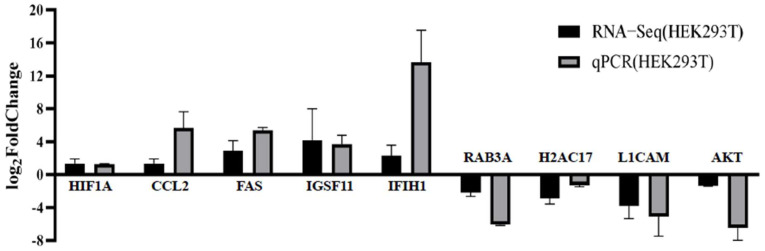
RT-qPCR Validation of gene expression. We verified the expression levels of nine genes (*HIF1A*, *CCL2*, *FAS*, *IGSF11*, *IFIH1*, *RAB3A*, *H2AC17*, *L1CAM*, and *AKT*) using RT-qPCR in HEK293T cells after A5L protein expression. RNA expression levels were normalized to *profilin1*. Error bars represent the standard deviation (SD) from at least three independent experiments.

To validate the accuracy and reliability of the RNA-Seq data, RT-qPCR was employed to identify some DEGs. The real-time qPCR primers are shown in Table 1. We transfected pCAGGS-HA-A5L into HEK293T cells. Nine genes (*HIF1A*, *CCL2*, *FAS*, *IGSF11*, *IFIH1*, *RAB3A*, *H2AC17*, *L1CAM*, and *AKT*) were randomly selected for RT-qPCR analysis. The results showed that the expressions of *HIF1A*, *CCL2*, *FAS*, *IGSF11*, and *IFIH1* were upregulated, while the expressions of *RAB3A*, *H2AC17*, *L1CAM*, and *AKT* were downregulated in pCAGGS-HA-A5L-transfected cells (Figure 5). The similarity of RT-qPCR data and RNA-Seq data indicated that the data from transcriptome was reliable.

### 2.6. Interaction of MPXV A5L

**Figure 6:**
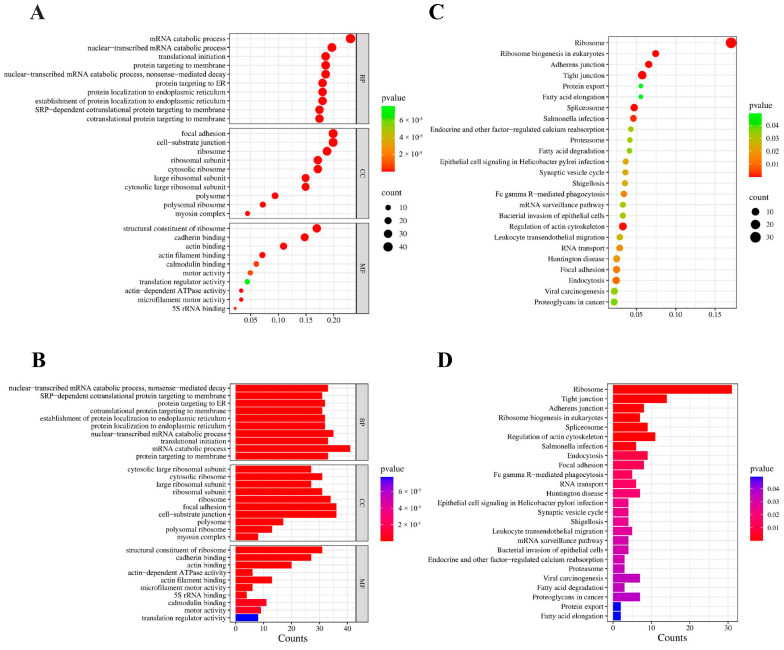
Functional enrichment analysis of proteins interacting with A5L. (**A**) The scatter plot and (**B**) the histogram of GO analysis. (**C**) The scatter plot and (**D**) the histogram of KEGG analysis. The horizontal axis indicates the gene ratio (**A**,**C**) or count (**B**,**D**). The vertical axis shows the top 30 or 25 most significance terms. Gene ratio: the ratio of the interacting proteins number to background proteins number. Count: the number of the interacting proteins. *p*-value: indicators of the significance of the term, the smaller the *p*-value.

The LC-MS/MS analysis was adopted to determine the interacting proteins of MPXV A5L protein for further study of its mechanism. Heterogeneous expression of MPXV A5L in HEK293T cells was immunoprecipitated using anti-HA beads, and the success of immunoprecipitation was confirmed by immunoblot (Figure 2D). The IP products were analyzed using LC-MS/MS, and the detailed results are summarized in Table 2. LC-MS/MS analysis identified a substantial number of ribosomal proteins (e.g., RPL8, RPL10A, and RPL7). The interaction between cytoskeleton proteins, including MYH9 and MYH10, with the MPXV A5L protein indicates that this protein may be involved in promoting the internalization of MPXV. Furthermore, our observations revealed a substantial presence of membrane proteins, including CALR, AMOT, and MYO6, suggesting that the MPXV A5L protein may play a critical role in regulating viral envelope formation and assembly. To further elucidate the biological functions and pathways of the identified proteins, we performed additional analysis using GO and KEGG enrichment methods. The GO analysis revealed that protein targeting to ER (GO:0045047) and translational initiation (GO:0006413) were prominent in BP, ribosome (GO:0005840) in the CC category, and actin binding (GO:0003779) and structural constituents of the ribosome (GO:0003735) were the most abundant GO terms in MF (Figure 6A,B). In the KEGG pathway enrichment analyses, the regulation of actin cytoskeletons (hsa04810), ribosomes (hsa03010), and spliceosomes (hsa03040) are significantly enriched (Figure 6C,D). The KEGG analysis revealed similar results between RNA-seq and mass spectrometry analysis, such as ribosome (hsa03010), RNA transport (hsa03013), and protein export (hsa03060) (Figure 4 and Figure 6).

## 3. Discussion

Over the past two years, MPXV has emerged as a zoonotic disease with significant implications for global public health [25,26]. Recently, the WHO Director-General declared the mpox outbreak a Public Health Emergency of international concern on 14 August 2024. Current case data indicate that MPXV outbreaks are becoming increasingly frequent, highlighting the ongoing risk posed by the genomic dynamics of DNA viruses for transmission and evolution within vulnerable populations [26,27]. With over 100,000 reported infections worldwide and no specific treatments available, there is an urgent need to investigate the gene functions and pathogenic mechanisms of MPXV [26,27]. MPOX poses a significant threat to global public health and security. Unfortunately, the underlying infection mechanisms of MPXV remain poorly understood. The research results on the MPXV core protein are limited. The core protein demonstrates notable conservation in poxviruses and shows potential as a novel avenue to target to develop pan-poxvirus vaccines or antiviral strategies. The membrane-free core of poxvirus consists of at least five abundant proteins, namely gene products of *A3L*, *A4L*, *A10L*, *L4R*, and *F17R* [28]. Except for F17R, these proteins are also associated with the incoming cytoplasmic core during the early stages of infection [28].

Given the aforementioned reasons, we investigated the characteristics and functions of the MPXV A5L protein using RNA-seq and LC-MS/MS. In this study, a total of 1473 DEGs were identified, including 911 upregulated and 562 downregulated genes, as well as 185 cellular proteins that interact with the MPXV A5L protein.

Viruses are obligate intracellular parasites that must rely on host cells to fulfill their genome replication requirements [29]. Viruses have evolved various mechanisms to redirect the host’s ribosomes toward their viral mRNAs [18]. As the virus hijacks the host translation machinery, the diversity of translating mRNAs decreases dramatically from tens of thousands of host mRNA species to only a few hundred viral mRNAs [30]. Similarly to all viruses, in order to ensure the efficient translation of virus proteins, VACV inhibits host protein synthesis and redirects the cellular translational machinery to the synthesis of viral proteins [31]; Newcastle disease virus (NDV) manipulates host translation mechanisms to facilitate viral replication [32]. A near-complete shutdown of host protein translation is induced by severe acute respiratory syndrome coronavirus (SARS-CoV) Nsp1 through a two-pronged strategy [33]. LC-MS/MS analysis revealed a significant quantity of ribosomal proteins interacting with MPXV A5L protein. These genes are associated with various GO terms related to ribosomal subunits, including cytosolic large ribosomal subunit (GO:0022625), cytosolic ribosome (GO:0022626), large ribosomal subunit (GO:0015934), and ribosome (GO:0005840). Some of the genes involved are RPL8, RPL10A, RPL7, RPL7A, RPL3, RPL4, RPS8, RPS23, RPL18, RPL15, RPL13, RPL5, and RPL6. RNA-seq analysis revealed that these differentially expressed genes (DEGs) are primarily associated with translation-related pathways such as the Ribosome pathway and the mTOR signaling pathway. In two additional mass spectrometry studies on MPXV proteins, the A23R protein was found to interact with various ribosomal proteins, whereas the F3L protein did not [5,26]. Both the A23R protein and A5L protein interact with a large number of ribosomal proteins but differ in their specific interactions. Specifically, A23R protein can bind to unique ribosomal proteins, including RPS15A, RPL17, and RPS16. The F3L protein interacts with host proteins involved in RNA splicing and protein translation, such as SNRNP70 and HNRNPH1 [5,26]. Similarly, the A5L protein also interacts with these host proteins, including SNRNP70 and HNRNPH2. KEGG pathway enrichment analyses revealed that the three MPXV proteins (A5L, A23R, and F3L) are involved in the spliceosome pathway and translation-related pathways. The observation suggested that the A5L protein has the ability to manipulate ribosomes in order to inhibit the host’s immune response and aid in viral transcription.

Poxviruses demonstrate an extraordinary degree of self-sufficiency and complexity in their replication and immune evasion strategies [18]. However, like all other viruses, poxviruses do not encode ribosomes and therefore remain entirely dependent on accessing the host translational machinery to synthesize viral proteins [34]. MPXV and other viruses impact host cell metabolism by dysregulating mTOR and the translation machinery (e.g., RACK1) to divert host resources toward the biosynthesis of viral components [18,25,35,36]. RACK1, which is part of the ribosome machinery, is phosphorylated by VACV to increase translation [25]. Alexis et al. showed that RACK1 is associated with RNA-binding proteins SERBP1 to facilitate Dengue virus (DENV) replication [37]. Avian leukosis virus (ALV) usurps the cellular SERBP1 protein to enhance its transcription and promote productive infections [38]. VACV kinase B1 phosphorylates the host small ribosomal protein RACK1, which enhances ribosome recruitment to poly(A) leader mRNAs [18,30,39]. SERBP1 is a member of the RG/RGG family of RNA-binding proteins, which regulates mRNA translation, but known protein patterns suggest additional regulatory functions [40,41,42,43]. The process of uncoating, whereby the proteinaceous core wall is breached and the DNA is released into the cytoplasm, is a crucial event in the temporal regulation of the viral genome cycle [44]. Once uncoating occurs and the viral core disassociates, early gene transcription ceases, presumably as a result of the dilution of the transcriptional machinery into the cytoplasm [44]. Our mass spectrometry data show that the SERBP1 and some ribosomal proteins interact with the MPXV A5L protein. This suggests that the MPXV A5L protein may enhance translation of viral proteins by affecting ribosomal function, warranting further investigation and confirmation.

The assembly of poxviruses starts with viral crescent [45]. This viral crescent grows until it forms a spherical structure called the immature virion. Maturation of these particles produces a brick-shaped virion, the mature virion [45]. Depending on the poxvirus genus, MVs may be enclosed by an additional membrane derived from the trans-Golgi, endosomal cisternae or plasma membrane to form wrapped virions (WVs) and transported to the cell periphery and exocytosed as extracellular enveloped virions (EEVs) [46]. Monkeypox is a member of the poxvirus and exists in two distinct infectious forms, the IMV and the EEV [3]. Mature virions possess three distinct layers: an outer envelope, an intermediate capsid-like layer, and an inner core [45]. The wrapping of IMVs by intracellular membranes requires the interaction between virus protein(s) on the IMV surface and the cytosolic face of the wrapping membranes [47]. The MPXV A5L protein is a homologue of the VACV A4L protein (Figure 1B). VACV A4L protein is most likely membrane associated through an interaction with an integral membrane protein(s) present in the innermost of the two membranes surrounding the IMV [48]. Indeed, in mature virions, the A4L protein binds to the outer domain of A10L, potentially tethering the palisade layer to the viral membrane [45]. This tethering could be necessary if confirmed, as it may explain how, during maturation, the spherical membrane previously stabilized by the immature virion scaffold collapses onto the core as it condenses [45]. Thus, A4L appears to be necessary for core assembly during the IV to MV transition [23]. According to LC-MS/MS analysis, MPXV A5L protein interacts with a variety of membrane proteins, such as SPTAN1, SPTBN1, CALR, and MYO6. Collectively, our findings highlight the potential role of MPXV A5L protein in virus assembly.

The replication process of poxviruses is distinctive and comprises several stages, beginning with attachment to host cells and culminating in the release of viral particles from infected cells [49]. MPXV, similar to other poxviruses, significantly disrupts cellular homeostasis by hijacking cytoskeleton organization to facilitate intracellular virion transport [25]. We observed the enrichments of DEGs in cytoskeleton-related pathways, such as myosin complex (GO:0016459), actin filament (GO:0005884), cell cortex (GO:0005938) and actin filament bundle (GO:0032432), etc. Additionally, interactions were also observed between the A5L protein and cytoskeletal proteins, including MYH9, MYH10, AMOT, etc. MYH9, also known as non-muscle myosin heavy chain 9, was first identified as a motor protein involved in cell migration, adhesion, and morphogenesis [50]. Complete virion internalization involves interactions between viral proteins and cellular receptors, as well as clathrin-mediated endocytosis facilitated by MYH9, an essential factor [50]. Studies have demonstrated that herpes simplex virus-1 (HSV-1), thrombocytopenia syndrome virus (SFTSV), Epstein–Barr virus (EBV), and Porcine reproductive and respiratory syndrome virus (PRRSV) rely on MYH9 as a crucial factor for infecting permissive cells [50,51,52]. Additionally, AMOT plays a significant role as a key regulator in various physiologically relevant pathways and processes, including transcription (via the Hippo pathway), apoptosis, cytoskeleton dynamics, and tight junction (TJ) integrity [53]. In the absence of AMOT, most virions were arrested at a ’half-moon’ stage, characterized by a hemisphere of assembled Gag molecules distending the membrane without forming a complete spherical particle [54]. These results suggested that MPXV A5L protein might have the ability to hijack cytoskeleton organization to facilitate virus internalization and assembly.

Currently, there is limited research available on the pathogenesis and life cycle of the MPXV. The primary focus of current research efforts is on developing vaccines, antiviral drugs, and accurate diagnostic methods for MPOX. Our findings highlight the potential role of MPXV A5L protein in regulating host gene expression and facilitating virus internalization and assembly. In the future, these findings need to be validated using techniques such as pull-down assays and fluorescence resonance energy transfer (FRET).

## 4. Conclusions

The global spread of MPXV has underscored the need for a deeper understanding of MPOX disease, particularly in comparison to other poxviruses. Transcriptomic and proteomic analyses of viruses have significantly advanced our knowledge of epidemic and pandemic viruses, facilitating drug development. The findings highlight the crucial role of the monkeypox virus A5L protein in the viral lifecycle. In addition to its involvement in viral morphogenesis, this protein may also play roles in (1) promoting viral internalization and assembly and (2) modulating the host translation system.

## 5. Materials and Methods

### 5.1. Cell Culture

HEK293T cells were cultured in Dulbecco’s Modified Eagle’s Medium (DMEM) (Solarbio, Beijing, China) supplemented with 10% heat-inactivated fetal bovine serum (FBS) (ExCell Bio, Shanghai, China) at 37 °C in a humidified atmosphere containing 5% CO_2_. The HEK293T cells were obtained from the China Type Culture Collection.

### 5.2. Plasmids Construction Transfection

The p39 protein-encoding gene *A5L* was synthesized according to the sequence of the MPXV strain MPXV-USA_2022_MA001 (GenBank No. NC_063383.1) and the gene synthesized by Beijing Tsingke Biotech Co., Ltd., Beijing, China. Primers were designed according to the MPXV-*A5L* gene sequence. The MPXV-*A5L* gene was inserted into the EcoR I and Xho I sites of the pCAGGS-HA plasmid to generate the pCAGGS-HA-A5L construct. MPXV *A5L* coding sequence was amplified via PCR using the forward primer 5′-CCGGAATTCATGGATTTTTTTAACAAATTTAGTCAGGG-3′ (EcoR I site underlined) and the reverse primer 5′-CCGCTCGAGTTTCTGAAAACGTTCAAAAC-3′ (Xho I site underlined). The resulting clones were verified by DNA sequencing. Plasmid transfection was performed using jetPRIMER^®^ Transfection Reagent (PolyPlus, Illkirch-Graffenstaden, France) following the manufacturer’s instructions.

### 5.3. DNA Transfection

293T cells were seeded in 6-well plates at a density of 2.5 × 10^5^ cells per well or in 10 cm dishes at a density of 1 × 10^6^ cells per well the day before transfection. pCAGGS-HA-A5L plasmid was transfected into the designated cells accordingly. 24 h post-transfection, a portion of the transfected cells was lysed in Trizol (Vazyme, Nanjing, China) for RNA extraction and subsequent RT-qPCR. The remaining cells were lysed in RIPA buffer (Solarbio, Beijing, China) for further Immunoblot analyses and immunoprecipitation. Cells transfected with an empty vector (pCAGGS-HA) served as a negative control.

### 5.4. RNA-Seq and Data Processing

RNA-seq were performed as described previously [5]. RNA-seq was performed to compare gene expression profiles between pCAGGS-HA-A5L-transfected 293T cells and pCAGGS-HA (empty vector)-transfected 293T cells over a 24 h time period. Three randomly selected samples from each group were used to prepare RNA-seq libraries. Total RNA was extracted from each individual sample using Trizol reagent (Invitrogen, Waltham, MA, USA). The quality and quantity of the total RNA were assessed using a NanoDrop spectrophotometer and an Agilent 2100 Bioanalyzer (Thermo Fisher Scientific, Waltham, MA, USA). Subsequently, mRNA was isolated using poly-T oligo-attached magnetic beads and then fragmented in fragmentation buffer at an appropriate temperature. First-strand cDNA was synthesized using random hexamers, followed by second-strand cDNA synthesis. End repair was performed by adding A-Tailing Mix and RNA Index Adapters. The cDNA fragments were PCR amplified and purified using Ampure XP beads. The products were validated on an Agilent 2100 Bioanalyzer (Thermo Fisher Scientific, USA) for quality control. The cDNA libraries were generated using the NEBNext^®^ Ultra™ RNA Library Prep Kit for Illumina^®^ (NEB, Ipswich, MA, USA) and sequenced on the Illumina NovaSeq 6000 platform (Illumina, San Diego, CA, USA).

The RNA-seq analysis was conducted by Novogene Biotech. Raw sequences were quality-controlled and filtered using fastp software (v0.23.1). To obtain high-quality data for downstream analyses, raw reads were was filtered using SOAPnuke (v1.5.2) to remove primer sequences and low-quality reads. Q20, Q30 and GC content of the clean reads were calculated in this process. The clean reads were mapped onto the Homo sapiens reference genome (GRCh38) using Hisat2 (v2.0.4) software with default parameters. Bowtie2 (v2.2.5) was applied to align the clean reads to the reference genome and StringTie (v2.1.2) was used to calculate fragments per Kilobase of transcript per million fragments mapped (FPKM) values for each gene. The differential expression analysis was performed using the DESeq2 (v1.4.5) with a significance criterion of Padj ≤ 0.05 and |log2(FC)| ≥ 0.35. The functional enrichment of DEGs were analyzed by Phyper (https://en.wikipedia.org/wiki/Hypergeometric_distribution, accessed on 25 September 2024) to identify enriched GO terms and KEGG pathways with a screening criterion of *p* < 0.05. Heatmaps were generated using an online platform for data analysis and visualization (https://www.bioinformatics.com.cn, accessed on 29 September 2024).

### 5.5. Real-Time Quantitative PCR (RT-qPCR)

To validate the reliability of the RNA-Seq data, 9 DEGs (*HIF1A*, *CCL2*, *FAS*, *IGSF11*, *IFIH1*, *RAB3A*, *H2AC17*, *L1CAM*, and *AKT*) were arbitrarily selected for RT-qPCR analysis. Total RNA was extracted from these splenocytes with TRIzol reagent following the supplier’s instructions. cDNA synthesis was performed according to the manufacturer’s protocols. RT-qPCR was developed in a QuantStudio 5 Real-Time PCR system (Applied Biosystems, Waltham, MA, USA). Each RT-qPCR was conducted in triplicate in 20 μL of total reaction volume, containing 10 μL of Universal SYBR Green Master mix, 1 μL of cDNA, and a pair of gene-specific primers. The RT-qPCR conditions were as follows: a pre-incubation (95 °C, 30 s), 40 amplification cycles (95 °C, 10 s; 60 °C, 30 s), and a final extension (95 °C, 15 s; 60 °C, 60 s). The expression levels of transcripts were normalized to that of the β-actin gene. The fold change in the expression of each target gene was estimated using the 2^−ΔΔCt^ method. The real-time qPCR primers are shown in Table 1.

### 5.6. Immunoblots Analyses

Immunoblots were performed as described previously [26]. Briefly, the cells were washed with PBS and lysed with RIPA Lysis Buffer. The lysates and protein samples were mixed with SDS sample loading buffer, boiled for 10 min, separated by SDS-PAGE, and transferred to polyvinylidene fluoride membranes (Millipore, Burlington, MA, USA). After blocking, the membranes were probed with the primary antibodies, followed by incubation with horseradish peroxidase-conjugated secondary antibody. The target proteins were visualized by using MonPro ECL Ultrasensitive Substrate Pro (Monad, Wuhan, China).

### 5.7. LC-MS/MS and Data Processing

LC-MS/MS were performed as described previously [26]. Briefly, HEK293T cells transfected with the pCAGGS-HA-A5L vector or the control vector were lysed in RIPA buffer (Solarbio, Beijing, China) supplemented with protease and phosphatase inhibitors. Cell lysates were incubated with anti-HA-agarose beads (Sigma Aldrich, Darmstadt, Germany) at 4 °C overnight. The beads were subsequently collected by centrifugation and washed with PBS before LC-MS/MS analysis. LC-MS/MS experiments and data analysis were conducted by Oebiotech. In brief, the proteins bound to the beads were denatured with 8 M urea in 50 mM NH_4_HCO_3_. The protein samples were sequentially reduced with dithiothreitol (DTT) (final concentration, 10 mM) at 55°C for 30 min, alkylated with iodoacetamide (final concentration, 15 mM), enzymatically hydrolyzed with trypsin overnight, and desalted with C18 columns. Peptides were analyzed on a Q Exactive-HF mass spectrometer (Thermo Scientific, USA) coupled with an Easy-nLC 1200 (Thermo Scientific, USA). The MS/MS signals were then processed against the National Center for Biotechnology Information (NCBI, Bethesda, MD, USA) protein database by using the Mascot Server (Matrix Science, Boston, MA, USA). Proteins with a ratio of abundance (A5L group/Control group) > 4 and score > 0 are considered to interact with A5L proteins. High-confidence peptides were selected and listed in Table 2. The protein “score” represents the standard score, which is the cumulative protein score obtained by summing the ion scores of the unique peptides. A higher score signifies greater confidence in identification. The pathway analysis was conducted using KOBAS (https://bioinfo.org/kobas/, accessed on 28 September 2024). The GO and KEGG pathways of *p* values less than 0.05 were considered significant and highly significant.

### 5.8. Statistical Analysis

Data were analyzed using GraphPad Prism 9.0 software to determine statistical significance. Inter-group statistical differences were assessed using Student’s *t*-test. *p* < 0.05 was considered statistically significant.

## Figures and Tables

**Table 1:** The primer sets were used for real-time qPCR analysis.

Gene	Sequence
(Human) HIF1α-F	TTTTGGCAGCAACGACACAG
(Human) HIF1α-R	GCGTTTCAGCGGTGGGTAAT
(Human) CCL2-F	TCAAACTGAAGCTCGCACTCT
(Human) CCL2-R	GGGGCATTGATTGCATCTGG
(Human) FAS-F	GTACGGAGTTGGGGAAGCTC
(Human) FAS-R	TTGATGTCAGTCACTTGGGCA
(Human) IGSF11-F	CTCTCTCTGCACGGTGTTGC
(Human) IGSF11-R	CATTGGAGAGAGGAGTGACCA
(Human) IFIH1-F	TCCATCGTTTGAGAACGCTCAT
(Human) IFIH1-R	CTGCAGCAGCAATCCGGT
(Human) RAB3A-F	AAAAGTCACCGCCGCTAGG
(Human) RAB3A-R	CTGCTGTTGCCGATGATGAG
(Human) H2AC17-F	TAGGCCACAGGTCGTTTTACC
(Human) H2AC17-R	CGTAGTTGCCCTTGCGGA
(Human) L1CAM-F	AGGTCCCTGGAGAGTGACAA
(Human) L1CAM-R	TACTGGCCAATGAACGAACCA
(Human) AKT-F	CCACACACTCACCGAGAACC
(Human) AKT-R	ACAGGTGGAAGAACAGCTCG
(Human) profilin1-F	ACGCCTACATCGACAACCTC
(Human) profilin1-R	CCTCAGCTGGCGTGATGT

**Table 2:** The list of interacting protein.

Accession	Score Sequest	Gene Symbol	Abundances (Control)	Abundances (A5L)	Fold
P55795	54.53	HNRNPH2		23,845,270.8	
Q9NSB2	31.28	KRT84		70,055,553.6	
P14649	21.06	MYL6B		53,201,279.2	
P62879	15.98	GNB2		21,537,638.7	
P12814	10.43	ACTN1		12,930,232	
Q9UHB6	7.13	LIMA1		54,735,281.5	
Q15027	6.55	ACAP1		78,746,156.2	
Q9BY77	5.8	POLDIP3		24,037,837.6	
Q96EY4	5.36	TMA16		17,209,636	
P35611	4.67	ADD1		22,683,268.1	
P22695	4.57	UQCRC2		7,952,299.99	
Q02241	4.16	KIF23		17,128,696.1	
O00159	3.82	MYO1C		30,334,815.4	
P08758	2.96	ANXA5L		11,660,744.3	
P34897	2.82	SHMT2		18,363,483.3	
O75251	2.74	NDUFS7		15,064,159.9	
Q13523	2.72	PRPF4B		10,888,648	
Q9BXS6	2.67	NUSAP1		18,715,571.1	
Q01650	2.62	SLC7A5L		16,038,306.4	
O15479	2.55	MAGEB2		38,857,514.5	
Q9UBM7	2.55	DHCR7		25,483,125.7	
Q13823	2.41	GNL2		20,441,894.6	
O94973	2.32	AP2A2		22,010,896.8	
P17028	2.32	ZNF24		7,071,781.83	
Q52LG2	2.29	KRTAP13-2		22,968,729.9	
P60953	2.2	CDC42		12,173,080.3	
P08174	2.2	CD55		47,053,652.5	
P27797	2.18	CALR		22,827,932.9	
Q9UHI6	2.13	DDX20		7,576,214.22	
Q9NWT1	2.06	PAK1IP1		11,007,573.8	
Q8WXE9	1.99	STON2		24,510,051.4	
Q9BQG1	1.98	SYT3		18,873,985.1	
P14210	1.94	HGF		19,177,626.6	
P49257	1.93	LMAN1		24,788,012.4	
Q9UQ88	1.91	CDK11A		8,636,393.03	
Q15005	1.9	SPCS2		5,468,154.27	
Q15323	1.88	KRT31		60,750,663.2	
P62330	1.88	ARF6		28,647,802.5	
P33992	1.87	MCM5		7,625,153.14	
Q96S97	1.86	MYADM		20,177,422.3	
Q15046	1.86	KARS1		9,191,269.95	
O00178	1.83	GTPBP1		26,055,539.3	
Q9Y490	1.79	TLN1		5,969,742.46	
Q96M91	1.77	CFAP53		76,472,193.3	
Q15654	1.76	TRIP6		17,252,038.1	
Q9P2M7	1.73	CGN		12,664,620.7	
O75915	1.68	ARL6IP5		31,787,231.5	
Q93008	1.68	USP9X		7,286,550.55	
P35241	1.65	RDX		19,958,722.5	
Q9H307	1.63	PNN		6,374,994.83	
P48200	1.63	IREB2		11,872,727.6	
Q96QK1	1.61	VPS35		13,607,837.2	
Q96A26	1.61	FAM162A		19,597,430.9	
Q15056	1.61	EIF4H		6,669,397.13	
P35579	1106.8	MYH9	7.6 × 10^9^	3.8008 × 10^10^	4.997828
P19338	393.64	NCL	4.32 × 10^9^	2.1659 × 10^10^	5.010884
P35580	718.97	MYH10	2.3 × 10^9^	1.0792 × 10^10^	4.686061
Q4VCS5	320.95	AMOT	1.7 × 10^9^	7,871,429,678	4.636524
P62917	185.85	RPL8	1.78 × 10^9^	7,114,210,016	4.003225
P62906	121.24	RPL10A	8.49 × 10^8^	5,080,928,660	5.987087
P18124	119.19	RPL7	7.35 × 10^8^	5,258,908,054	7.157648
P62424	118.83	RPL7A	5.53 × 10^8^	3,868,490,859	6.998979
Q13813	109.9	SPTAN1	3.8 × 10^8^	1,852,745,858	4.874794
P39023	105.74	RPL3	4.8 × 10^8^	4,126,009,236	8.593177
P36578	104.96	RPL4	5.6 × 10^8^	3,130,805,766	5.590338
Q8NC51	100.27	SERBP1	5.29 × 10^8^	2,247,852,488	4.252472
P62241	85.38	RPS8	9.77 × 10^8^	6,094,990,507	6.237919
P62266	84.37	RPS23	3.6 × 10^8^	1,797,616,211	4.998203
Q07065	71.21	CKAP4	1.68 × 10^8^	794,929,264	4.719978
P62753	70.84	RPS6	5.11 × 10^8^	2,638,295,624	5.158366
Q07020	69.52	RPL18	4.68 × 10^8^	3,943,925,150	8.431103
P61313	66.41	RPL15	4.07 × 10^8^	3,923,911,952	9.630158
P26373	65.09	RPL13	2.44 × 10^8^	3,306,516,447	13.55998
Q02878	63.03	RPL6	3.75 × 10^8^	1,579,582,645	4.213828
P46777	62.53	RPL5	3.06 × 10^8^	1,550,140,466	5.058458
P61353	62.24	RPL27	3.83 × 10^8^	3,286,606,950	8.57408
P62888	55.07	RPL30	3.32 × 10^8^	1,832,429,225	5.517408
Q9UNX3	52.15	RPL26L1	4,933,386	23,405,671.6	4.744343
P27635	51.33	RPL10	2.26 × 10^8^	943,176,519	4.177723
Q02543	51.22	RPL18A	4.56 × 10^8^	2,303,106,278	5.051142
Q9Y3U8	49.59	RPL36	5.96 × 10^8^	3,349,708,990	5.618173
P62854	45.66	RPS26	1.99 × 10^8^	1,494,638,724	7.527102
Q9UM54	45.24	MYO6	63,204,009	734,670,570	11.6238
Q92522	44.99	H1-10	1.63 × 10^8^	678,302,362	4.173634
P46776	43.04	RPL27A	3.6 × 10^8^	1,757,091,613	4.884333
Q01082	42.52	SPTBN1	70771524	566,802,222	8.008902
P62910	41.13	RPL32	2.87 × 10^8^	1,269,884,943	4.431853
P53621	41.12	COPA	1.46 × 10^8^	724,068,452	4.953142
P46087	36.96	NOP2	1.07 × 10^8^	1,173,149,722	10.98216
Q08170	32.18	SRSF4	2.52 × 10^8^	1,535,299,732	6.084086
Q16643	30.41	DBN1	31,211,853	767,446,859	24.58831
P61513	28.38	RPL37A	2.3 × 10^8^	1,238,388,622	5.394924
P31946	27.98	YWHAB	7,652,528	77,359,209.5	10.10898
O94832	25.9	MYO1D	2,752,137	357,771,870	129.9978
Q9NQ29	25.17	LUC7L	5,746,320	23,316,664.9	4.057669
O43795	25.12	MYO1B	47,966,804	449,804,703	9.377417
P12277	23.32	CKB	79,100,780	350,631,774	4.432722
E9PAV3	22.7	NACA	54,653,562	494,198,804	9.04239
P46779	21.98	RPL28	97,888,915	544,056,692	5.557899
Q8N3R9	19.26	PALS1	20,378,481	154,737,012	7.593157
P08754	19.24	GNAI3	60,146,303	479,782,974	7.976932
P50914	19.15	RPL14	1.78 × 10^8^	1,393,870,977	7.83708
P40429	19.01	RPL13A	1.89 × 10^8^	1,509,570,649	7.967573
P49207	18.93	RPL34	3.87 × 10^8^	2,066,637,763	5.346116
Q9UMS4	17.92	PRPF19	1.35 × 10^8^	661,151,564	4.913278
P42766	16.42	RPL35	1.73 × 10^8^	828,704,701	4.791414
O95793	16.04	STAU1	34,599,041	146,473,177	4.233446
Q9P2E9	15.7	RRBP1	36,221,922	171,298,674	4.729144
Q15149	14.76	PLEC	13,070,259	222,413,318	17.01675
Q9H7B2	14.29	RPF2	12565969	145,092,561	11.54647
P61006	14.02	RAB8A	2,400,302	16,719,589.6	6.965618
Q13610	13.96	PWP1	18,865,848	141,208,069	7.484852
P62633	13.76	CNBP	23,925,131	176,695,010	7.385331
O43707	13.27	ACTN4	24,205,268	136,102,708	5.622855
P47914	13.01	RPL29	5.9 × 10^8^	2,512,280,276	4.256594
P13987	12.93	CD59	33,261,676	215,455,509	6.47759
Q8TDN6	12.65	BRIX1	10,376,578	168,688,087	16.25662
Q04637	10.76	EIF4G1	29,253,382	146,869,232	5.02059
P08621	9.91	SNRNP70	23,918,842	160,794,203	6.722491
Q9BZE4	9.69	GTPBP4	30,334,456	218,282,534	7.195861
Q9Y678	9.66	COPG1	14,504,055	58,685,143.1	4.04612
Q8NE71	9.47	ABCF1	9,205,467	48,876,803.4	5.309541
Q07157	9.45	TJP1	9,895,750	53,372,921.9	5.39352
Q9BQG0	8.69	MYBBP1A	26,923,852	147,077,400	5.462718
O43242	8.51	PSMD3	24,866,727	121,259,423	4.876372
P49756	8.1	RBM25	12,400,093	88,151,215.8	7.108916
O75400	7.98	PRPF40A	27,402,617	116,759,966	4.260906
Q14684	7.93	RRP1B	69,654,704	353,008,391	5.067976
P08243	7.75	ASNS	16,356,520	223,274,864	13.65051
Q9Y2X3	7.6	NOP58	3,452,465	59,794,539.1	17.31938
P05455	7.34	SSB	13,230,934	94,151,395.8	7.116005
Q01105	7.24	SET	32,879,772	135,036,667	4.106983
Q9NW13	7	RBM28	8,323,326	100,335,155	12.05469
P42696	6.65	RBM34	8,732,097	40,401,561.4	4.626788
Q5JWF2	6.47	GNAS	4,735,516	74,347,665.1	15.70001
Q8IY81	6.47	FTSJ3	3,963,930	44,935,915.6	11.3362
Q9NX58	6.36	LYAR	11718590	65,237,865.7	5.567041
P63000	5.98	RAC1	18,650,276	99,502,685.4	5.335186
P61221	5.79	ABCE1	15,830,286	150,435,816	9.503039
P62891	5.71	RPL39	29,916,276	163,424,396	5.462725
Q96CW1	5.68	AP2M1	13,251,519	61,822,112	4.665285
P40939	5.47	HADHA	13,623,453	67,584,883.9	4.960922
Q9BRJ6	5.46	C7orf50	19,859,188	95,007,628.2	4.784064
Q9H9B4	5.06	SFXN1	9,005,179	216,387,269	24.0292
Q86VP6	5.04	CAND1	8,920,870	66,555,261.6	7.460625
Q6P2Q9	5.03	PRPF8	2,161,177	38,374,317.9	17.75621
P46940	4.91	IQGAP1	7,938,699	109,080,082	13.7403
Q9Y295	4.81	DRG1	4747110	19,024,856.7	4.007671
Q13642	4.8	FHL1	2,702,271	45,495,752.3	16.83612
Q9BUJ2	4.76	HNRNPUL1	9,271,129	58,503,614.9	6.310301
O14654	4.56	IRS4	6,215,177	50,639,493.4	8.147716
O60763	4.52	USO1	4,183,194	24,127,383.2	5.767694
O75643	4.44	SNRNP200	9,569,121	70,538,432.2	7.371464
O43592	4.27	XPOT	5,525,361	30,283,421.8	5.480805
Q86UP2	4.24	KTN1	12,269,741	77,750,053.4	6.336731
Q9P2J5	4.2	LARS1	18,835,668	82,710,799.8	4.39118
Q86VM9	3.96	ZC3H18	2,471,121	24,366,387.4	9.860458
O76094	3.95	SRP72	9,620,050	41,076,561.6	4.269891
Q9UKD2	3.95	MRTO4	7,057,619	78,872,065.8	11.17545
Q14697	3.56	GANAB	9,795,123	61,834,988.6	6.312834
P53396	3.37	ACLY	6,139,189	28,311,705.6	4.611636
Q01780	2.86	EXOSC10	3,204,284	16,656,062.6	5.198061
Q86UE4	2.71	MTDH	8,321,409	35,187,694.4	4.228574
P63151	2.62	PPP2R2A	5,711,416	36,989,877.6	6.476481
Q9NZM5	2.61	NOP53	5,430,802	30,995,616	5.707374
O75369	2.57	FLNB	2,276,534	61,851,286.7	27.16906
Q8NI35	2.39	PATJ	3,567,723	48,585,809.7	13.61816
Q03701	2.18	CEBPZ	4,374,025	17,743,194.8	4.056491
Q92896	2.18	GLG1	800,199.9	4,717,955.06	5.89597
Q9Y4P3	2.17	TBL2	5,922,978	43,983,036.8	7.425832
Q16630	2.16	CPSF6	11,481,576	50,123,009.6	4.365517
Q9H4G4	2.15	GLIPR2	5,717,304	30,078,632.4	5.260982
O95831	2.13	AIFM1	3,878,012	45,361,458.8	11.69709
P20290	2.08	BTF3	6,736,184	28,957,538.5	4.298805
Q92558	2.01	WASF1	1,504,043	7,875,298.01	5.236086
P13010	1.96	XRCC5	1,067,594	24,486,127.9	22.93581
Q8NB91	1.91	FANCB	4.13 × 10^9^	1.7678 × 10^10^	4.283121
Q9H0A0	1.9	NAT10	5,435,425	42,088,224.7	7.743318
P78347	1.9	GTF2I	4,062,462	57,972,505.3	14.27029
P06493	1.83	CDK1	9,830,338	97,019,514.9	9.869398
O00231	1.76	PSMD11	6,285,229	36,586,283.9	5.820995
Q8TEX9	1.74	IPO4	3,142,446	18,886,047	6.009983
Q14839	1.73	CHD4	5,117,228	22,926,902	4.480337
Q5T8P6	1.63	RBM26	3,233,657	14,625,610.8	4.522932

## Data Availability

The original contributions presented in the study are publicly available. This RNA-seq data can be found here: [NCBI/PRJNA1181317]. The mass spectrometry proteomics data have been deposited to the ProteomeXchange Consortium (https://proteomecentral.proteomexchange.org) via the iProX partner repository with the dataset identifier PXD057872.

## Abbreviations

MPOX	Monkeypox
MPXV	Monkeypox virus
VARV	Variola virus
CPXV	Cowpox virus
VACV	Vaccinia virus
RNA-seq	RNA sequencing
LC-MS/MS	Liquid chromatography-tandem mass spectrometry
HEK293T Cell	Human Embryonic Kidney 293 Cells
GO	Gene Ontology
KEGG	Kyoto Encyclopedia of Genes and Genomes
WHO	World Health Organization
PHEIC	Public Health Emergency of International Concern
ORFs	open reading frames
IMV	Intracellular mature virion
IV	Intracellular virions
MV	Mature virions
DEGs	differentially expressed genes
Co-IP	Co-immunoprecipitation
WCL	Whole-cell lysate
BP	biological process
CC	cellular component
MF	molecular function
NDV	Newcastle disease virus
SARS-CoV	Severe acute respiratory syndrome coronavirus
DENV	Dengue virus
ALV	Avian leukosis virus
WV	Wrapped virions
EEV	Extracellular enveloped virion
HSV-1	Human simplex virus-1
SFTSV	Thrombocytopenia syndrome virus
EBV	Epstein–Barr virus
PRRSV	Porcine reproductive and respiratory syndrome virus
FPKM	fragments per kilobase of transcript per million mapped reads
DTT	dithiothreitol
NCBI	National Center for Biotechnology Information
